# Supplementary material for: Potential for future reductions of global GHG and air pollutants from circular waste management systems
Source: Nat Commun. 2022 Jan 10;13:106. doi: 10.1038/s41467-021-27624-7 (PMC8748894; doi:10.1038/s41467-021-27624-7)
Supplement: Supplementary file 1 — Supplementary Information [file 41467_2021_27624_MOESM1_ESM.pdf]

Supplementary Information to :

Potentials for future reductions of global GHG and air pollutants from circular waste management systems

Adriana Gómez-Sanabria [1,2*], Gregor Kiesewetter[1], Zbigniew Klimont[1], Wolfgang Schoepp[1] & Helmut Haberl[2]

[1] Pollution Management Research Group, Energy, Climate and Environment Program, International Institute for Applied Systems Analysis, Laxenburg, Austria

[2] Institute of Social Ecology, University of Natural Resources and Life Sciences, Vienna, Austria

Corresponding author

Correspondence to: Adriana Gómez-Sanabria. Email: gomezsa@iiasa.ac.at

Contents

S1. Waste generation in urban and rural areas by region 2

S2. Scenario Analysis. 5

S3. Comparison of emissions from MSW 8

S4. Description of the SSPS. 10

S5. Abbreviations 12

S6. GDP per capita and share of urban population. 13

S7. Description of the methodology to project municipal solid waste generation and composition. ... 15

S8. Rural-urban waste generation ratio 21

S9. Waste matrix in GAINS. 22

S10. MSW management narratives and regional aggregation. 22

S1. Waste generation in urban and rural areas by region

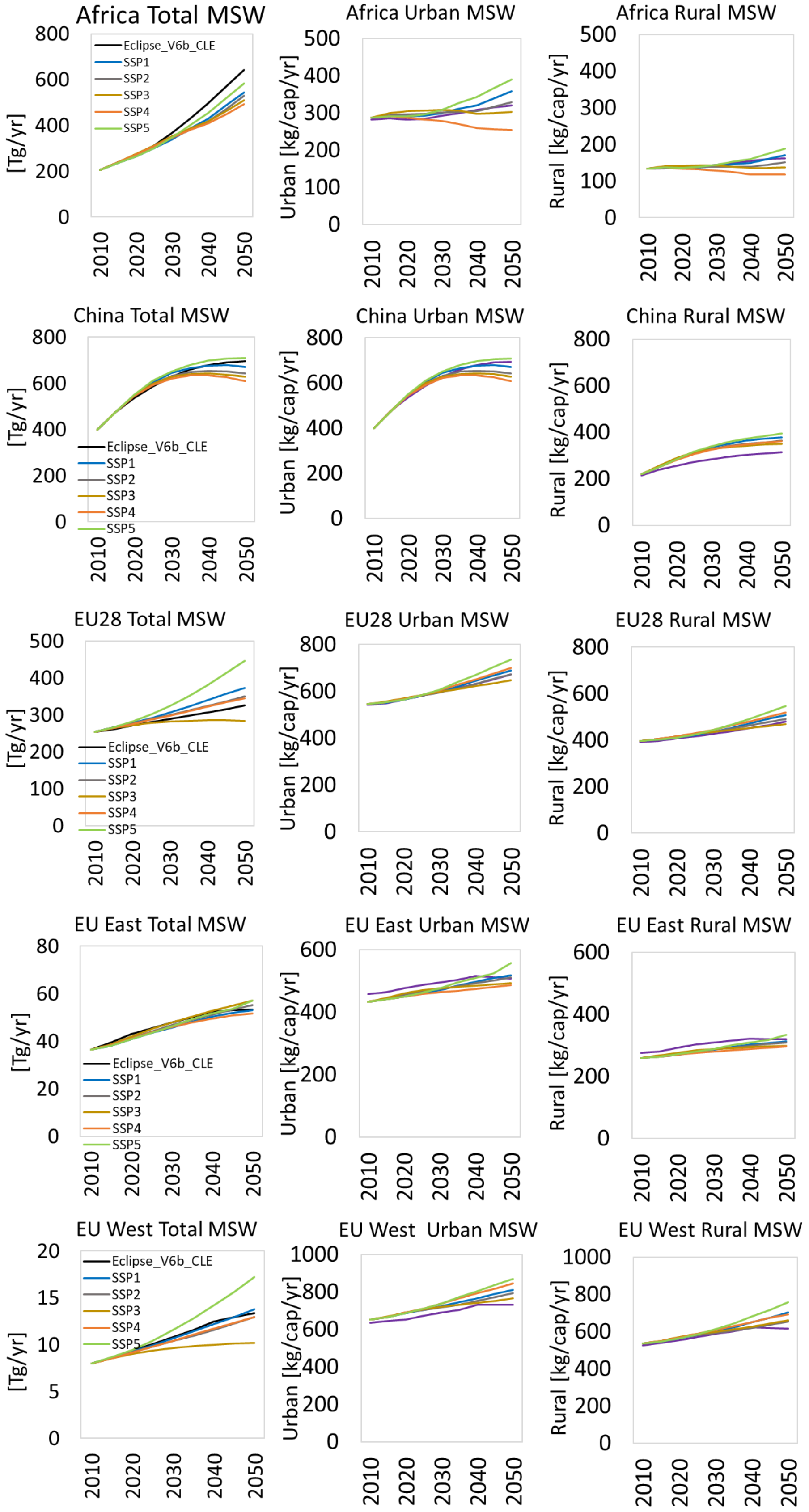

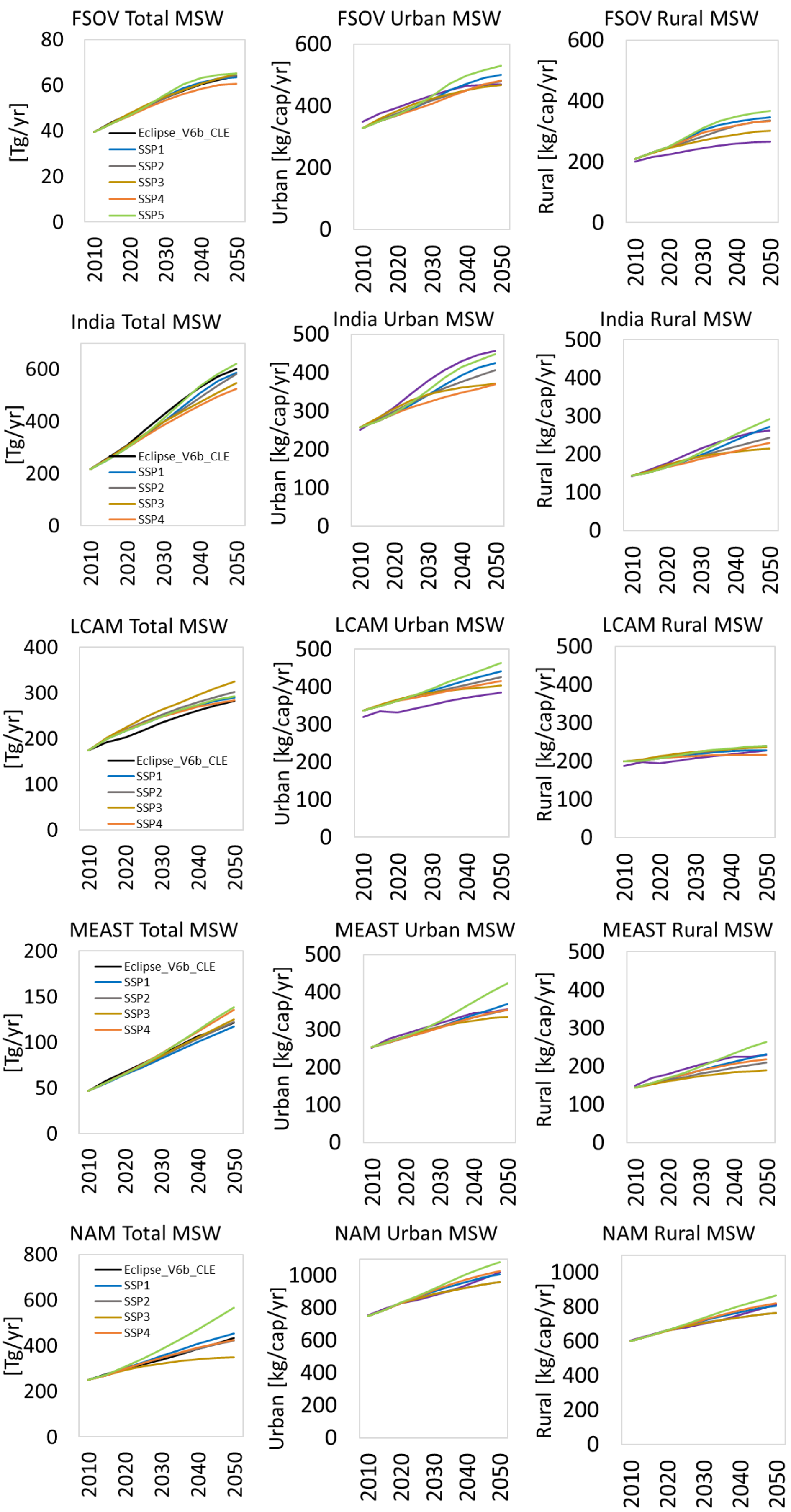
FSOV Total MSW
[Tg/yr]
0
20
40
60
80
Eclipse_V6b_CLE
SSP1
SSP2
SSP3
SSP4
SSP5
2010
2020
2030
2040
2050
FSOV Urban MSW
Urban [kg/cap/yr]
0
200
400
600
FSOV Rural MSW
Rural [kg/cap/yr]
0
200
400
600
India Total MSW
[Tg/yr]
0
200
400
600
Eclipse_V6b_CLE
SSP1
SSP2
SSP3
SSP4
India Urban MSW
Urban [kg/cap/yr]
0
100
200
300
400
500
India Rural MSW
Rural [kg/cap/yr]
0
100
200
300
400
500
LCAM Total MSW
[Tg/yr]
0
100
200
300
400
Eclipse_V6b_CLE
SSP1
SSP2
SSP3
SSP4
LCAM Urban MSW
Urban [kg/cap/yr]
0
100
200
300
400
500
LCAM Rural MSW
Rural [kg/cap/yr]
0
100
200
300
400
500
MEAST Total MSW
[Tg/yr]
0
50
100
150
200
Eclipse_V6b_CLE
SSP1
SSP2
SSP3
SSP4
MEAST Urban MSW
Urban [kg/cap/yr]
0
100
200
300
400
500
MEAST Rural MSW
Rural [kg/cap/yr]
0
100
200
300
400
500
NAM Total MSW
[Tg/yr]
0
200
400
600
800
Eclipse_V6b_CLE
SSP1
SSP2
SSP3
SSP4
NAM Urban MSW
Urban [kg/cap/yr]
0
200
400
600
800
1000
NAM Rural MSW
Rural [kg/cap/yr]
0
200
400
600
800
1000

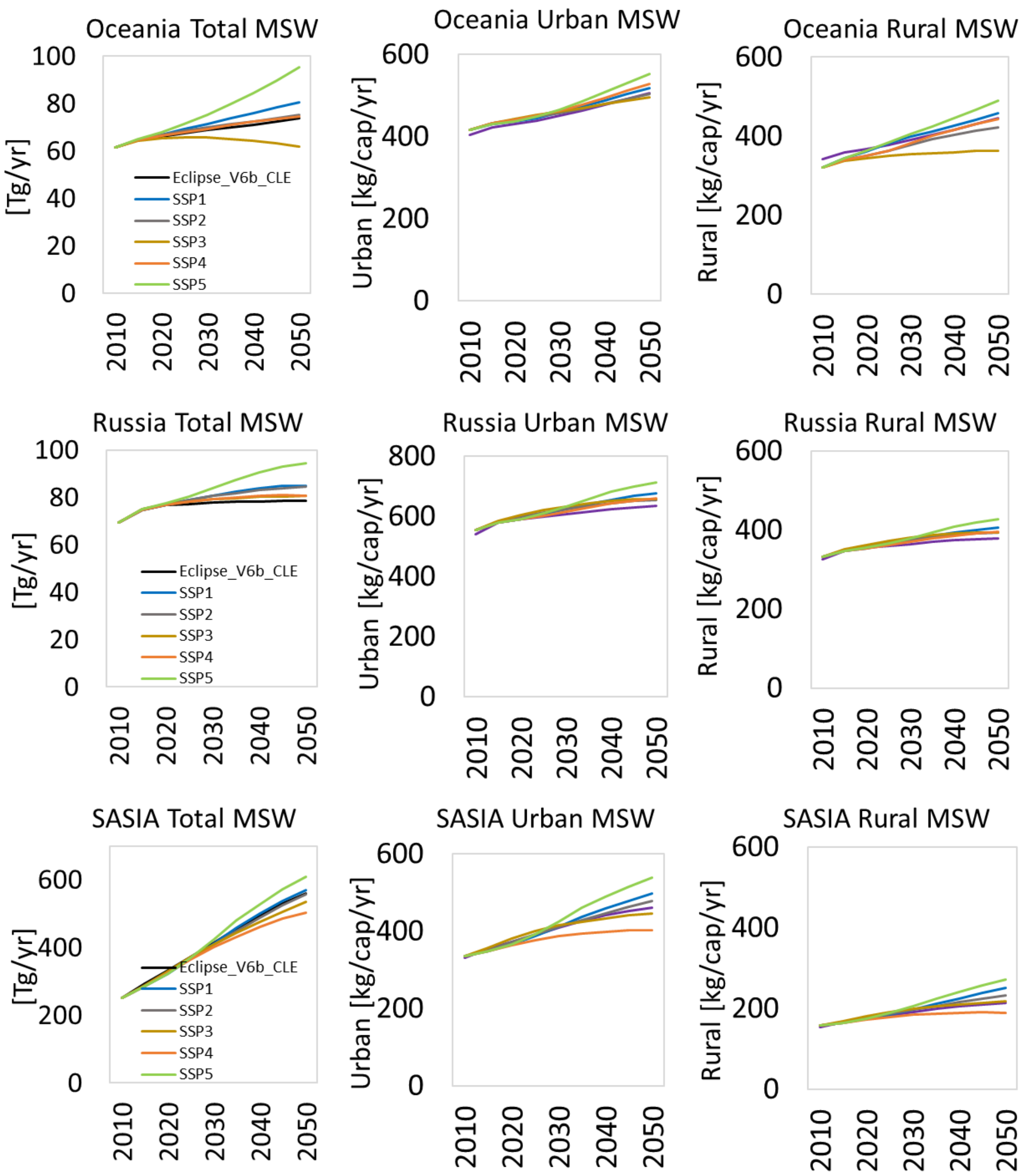

Fig. S1. MSW generation in urban and rural areas by region – scenario

S2. Scenario Analysis.

The following section presents an analysis of the mitigation scenarios along with regional figures. Furthermore, Table S1 presents a summary of MSW generation, management, and related emissions for 2030 and 2050.

Table S1. Global estimations of MSW, CH4, particulate matter and air pollutants for the baseline scenarios (CLE) and the mitigation scenarios (MFR).

	SSP1						SSP2					
	SSP1_CLE			SSP1_MFR			SSP2_CLE			SSP2_MFR		
	2030	2040	2050	2030	2040	2050	2030	2040	2050	2030	2040	2050
MSW generation (Tg)	3048	3517	3901	2421	2771	3059	3025	3423	3801	3025	3423	3801
MSW dumpsites/non-sanitary landfills (Tg)	1011	1180	1327	88	0	0	965	1099	1239	534	131	0
MSW openly burned (Tg)	566	680	779	21	0	0	568	666	767	289	46	0
CH4 (Gg)	36991	45861	53692	32335	17531	6706	36633	44966	51862	38212	39509	22559
CO (Gg)	14352	16112	17406	574	12	14	14937	16657	18206	7794	1184	17
CO2 (Gg)	200145	235671	267753	91708	175940	198494	201819	230096	259303	145314	164740	313547
NOX (Gg)	557	654	735	151	189	213	565	648	730	337	214	257
PM_2_5 (Gg)	3012	3387	3665	118	1	1	3133	3497	3828	1629	245	1
PM_BC (Gg)	227	256	277	9	0	0	236	264	289	123	19	0
PM_OC (Gg)	1795	2014	2175	71	0	0	1869	2083	2276	975	147	0
SO2 (Gg)	110	129	145	16	18	21	111	128	144	60	25	25
VOC (Gg)	2886	3239	3498	115	2	2	3004	3349	3660	1568	238	3
	SSP3						SSP4					
	SSP3_CLE			SSP3_MFR			SSP4_CLE			SSP4_MFR		
	2030	2040	2050	2030	2040	2050	2030	2040	2050	2030	2040	2050
MSW generation (Tg)	2994	3302	3579	2994	3302	3579	2990	3330	3602	2990	3330	3602
MSW dumpsites/non-sanitary landfills (Tg)	917	1010	1110	533	139	0	994	1123	1235	554	152	0
MSW openly burned (Tg)	576	659	742	391	97	0	554	633	706	348	79	0
CH4 (Gg)	36192	43804	49434	37441	38763	23696	37050	45656	52404	38309	38352	22791
CO (Gg)	15808	17617	19251	12035	3200	16	14004	15028	15767	9840	2391	16
CO2 (Gg)	203234	223929	242499	164259	172697	290713	193053	213202	229575	153296	170134	283600
NOX (Gg)	576	647	712	433	251	238	543	605	656	388	234	233
PM_2_5 (Gg)	3313	3694	4039	2513	665	1	2939	3158	3317	2056	497	1
PM_BC (Gg)	249	278	304	189	50	0	222	238	251	155	37	0
PM_OC (Gg)	1978	2205	2409	1507	400	0	1752	1879	1971	1232	298	0
SO2 (Gg)	114	129	143	82	34	23	107	119	129	71	30	23
VOC (Gg)	3179	3543	3871	2422	644	3	2816	3021	3169	1980	481	3
	SSP5						ECLIPSE_V6b					
	SSP5_CLE			SSP5_MFR			ECLIPSE_V6b_CLE			ECLIPSE_V6b_MFR		
	2030	2040	2050	2030	2040	2050	2030	2040	2050	2030	2040	2050
MSW generation (Tg)	3144	3758	4296	3144	3758	4296	3043	3536	3948	3043	3536	3948
MSW dumpsites/non-sanitary landfills (Tg)	1032	1234	1414	160	0	0	964	1129	1282	149	0	0
MSW openly burned (Tg)	579	717	832	43	0	0	576	704	811	38	0	0
CH4 (Gg)	37100	46520	55618	37003	23834	11064	36557	44634	52040	36443	23051	10881
CO (Gg)	14667	16946	18621	1151	18	21	15255	17640	19225	1092	16	18
CO2 (Gg)	209869	260156	308042	139975	317820	374100	201914	238015	268572	130963	286463	321425
NOX (Gg)	574	699	804	191	260	305	571	680	765	182	236	264
PM_2_5 (Gg)	3079	3563	3922	239	1	1	3199	3704	4041	226	1	1
PM_BC (Gg)	232	269	296	19	0	0	241	279	305	18	0	0
PM_OC (Gg)	1834	2118	2327	143	0	0	1909	2207	2404	135	0	0
SO2 (Gg)	113	137	157	22	25	30	113	135	152	21	23	26
VOC (Gg)	2949	3406	3741	231	3	3	3068	3547	3865	219	3	3

SSP1_MFR: The global adoption of the measure targeting the reduction of urban municipal food and plastic waste of 50% by 2030 reduces the global MSW generation by about 20% compared to the baseline (SSP1_CLE). Compared to SSP1_CLE, regions as Africa, China, SASIA, LCAM will experience a constant reduction of 30% per year on MSW generation between 2030 and 2050. India is expected to reduce MSW generation by 15% in 2030, reaching a maximum reduction of 18% in 2050.

The rest of the world is expected to have a steady reduction of about 18% per year until 2050. Collection rates in developing regions will reach the levels of the EU in both, urban and rural areas achieving rates of >= 95% already in 2030. The reduction of MSW couple with the implementation of MSW management policies at a global level as a consequence of technology transfer and capacity building in less favored countries, including rural areas, will result in a global reduction of MSW openly burned of 96 % in 2030 and close to 100% in 2050. Consequently, same reduction percentage of particulate matter and air pollutants emissions will be observed. This reduction in emissions will have a notorious positive impact on air quality and can potentially bring some climate benefits [1–3]. Thus, it will be possible to avoid the release of 218 Gg/yr BC in 2030 and 277 Gg/yr BC in 2050. The speedy implementation of anaerobic digestion to treat organic waste and the establishment of source separated MSW collection to increase the recycling of materials achieves MSW landfill/dumpsite reduction of 92% in 2030 and 97% in 2050 compared to the same years in the SSP1_CLE. The uniform improvement of MSW management systems at a global level reduces CH_4 emissions by 13% in 2030 and 88% in 2050 compared to the same years in SSP1_CLE. In 2050, CO_2 emissions from MSW will be 26% lower compared to the corresponding baseline (CLE). In 2035, it will be possible to eliminate air pollution from open burning of MSW.

SSP2_MFR: MSW generation is expected to be the same as in SSP2_CLE due to the absence of measures targeting its reduction. There is an improvement in the global waste management system at a global level, however, inequalities are observed in the developing countries. Though those countries start taking MSW waste management as an important point in the political agenda, the implementation of the MSW management strategies, although possible, is challenging. After 2030, the implementation of the adopted measures shows an improvement. As a result, 45% less MSW, equivalent to 534 Tg/yr ends up in non-sanitary landfills/dumpsites in 2030 compared to the same year in SSP2_CLE. The quantities of MSW openly burned can be reduce by about 49% in 2030. While this pathway does not really affect developed regions such as Europe (EU28 and EU West), North America and Oceania due to the maturity of the management systems, it certainly makes more difficult for the rest of the world to cope with the increasing quantities of MSW generation. Thus, CH4 emissions will increase 4% in 2030 and a maximum reduction of 57% will be observed in 2050 compared to the same years in SSP2_CLE. CO_2 emissions will increase by 25% in 2050. A maximum emission reduction of particulate matter and air pollutants of 48% is observed in 2030.

SSP3_MFR: This scenario depicts the lowest MSW generation quantities within the modelled scenarios, due to the little economic growth and slow urbanization. The improvement of MSW management systems is rather slow resulting from the lack of investment, international support, and education. While the developed world can continue enhancing the MSW systems within the circular economy framework, developing countries really struggle with the quantities of MSW generation, thus,

reaching the target of ~zero emissions from MSW management in 2050 is more than challenging. Particularly, rural areas start adopting strategies to improve MSW systems after 2030. Due to the slow adoption of measures, the maximum reduction of MSW ending in landfills/dumpsites and MSW open burned will be 55% and 32% in 2030, respectively. With the improvement of MSW management is still expected that 533 Tg/yr will end up in non-sanitary landfills/dumpsites in 2030. Thus, generating 3% more CH_4 emissions for the same year compared to the baseline. MSW openly burned will be reduced to 391 Tg/yr in 2030 and reach ~zero in 2050. After 2030 developing countries start to replicate measures from the developed world thus there is a decline in emissions towards 2050. In the SSP3_MFR scenario, CH_4 emissions are estimated to be 23696 Gg/yr in 2050 being the highest in all MFR scenarios.

SSP4_MFR: Similarly, to SSP3_MFR, this scenario reflects the inequalities between the high- and middle-income countries and the low-income countries. This is reflected in the pace and level of the adopted measures to deal with the amounts of MSW generated. While this socio-economic development does not really affect industrialized regions, certainly is difficult for developing countries to improve MSW, particularly in rural areas. The slow phase-in of circular MSW management still leaves 994 Tg/yr of MSW to end up in non-sanitary landfills/dumpsites in 2030. MSW openly burned will be reduced to 554 Tg/yr in 2030 and reach ~zero in 2050. CH_4 emissions will be rather the same in the SSP4_CLE and SSP4_MFR scenarios by 2030 while maximum reduction of particulate matter and air pollutants in the MFR will be 37% compared to their CLE counterparts. CH_4 emissions are expected to be reduced to 22791 Gg/yr in 2050. The reduction of particulate matter and air pollutants is proportional to the reduction of MSW openly burned. CO_2 are expected to increase to 283600 Gg/yr in 2050 which is 24% higher compared to the SSP4_CLE.

SSP5_MFR: As a result of the increase in global income MSW generation reaches the highest quantities among the modelled scenarios. However, due to the MSW technology transfer and capacity building in the less favored countries, it is possible to drastically improve MSW systems, in both urban and rural areas, and hence reduce emissions. Still, solutions are focused only on technical approaches and end-of-pipe solutions and measures targeting reduction of MSW generation at source are lacking. Regions as Africa, India, South Asia and Latin America and the Caribbean quickly adopt anaerobic digestion to treat organic MSW and increase recycling rates of recyclables. Incineration plays an important role in the treatment of refuse. Even with the fast implementation of the circular MSW management systems, we estimate that 160 Tg/yr of MSW will still end up in non-sanitary landfills/dumpsites in 2030. 43 Tg/yr of MSW are expected to be open burned in 2030. CH_4 will be reduced to 80% in 2050 compared to SSP5_CLE, i.e., 10881 Gg/yr CH_4 will be emitted in 2050. As MSW open burned is estimated to be reduced by 93% in 2030 compared to SSP5_CLE, particulate matter and air pollutants from this source are reduced at the same level. We estimate that towards 2050 MSW open burned could be close to totally avoidable and therefore emissions associated to this practice as well.

ECLIPSE_V6b_MFR: The development of this scenario is quite similar to the SSP5_MFR in which the focus is technological solutions. As MSW generation is a bit lower than in the SSP5, baseline emissions are also correspondingly lower. We estimate that 149 Tg/yr of MSW will still end up in non-sanitary landfills/dumpsites in 2030. 38 Tg/yr of MSW are expected to be open burned in 2030. CH_4 will be reduced to 79% in 2050 compared to ECLIPSE_V6b_CLE, i.e., 108881Gg CH_4 will be emitted in 2050. As MSW open burned is estimated to be reduced by 93% in 2030 compared to ECLIPSE_V6b_MFR, particulate matter and air pollutants from this source are reduced at a similar level.

S3. Comparison of emissions from MSW

Table S2. Studies assessing Global CH_4 emissions from waste Tg/yr.

Study	Sector	2010	2011	2012	2013	2014	2015	2050	Notes
EDGARv 4.3.2 [4]	Waste	37	38	37	37	38	38		Includes industrial waste and MSW
CMIP6 [5,6]	Waste	33	33	33	34	34	34		Includes industrial waste and MSW
Wiedinmyer et al., 2014[7]	MSW	4							Open burning of MSW
Eclipse_V5a [2]	MSW	35					30	57	
This study	MSW	29					30	49-55	Min 49 Tg for SSP3 and Max 55 Tg for SSP5. All other Scenarios in between this range
CMIP6 [5,6]	Total	371		381		388	388		
Höglund-Isaksson, 2020[8]	Total						344	450	Total global anthropogenic CH_4

Table S3. Studies assessing Global CO_2 emissions from waste Tg/yr.

Study	Sector	2010	2011	2012	2013	2014	2015	2050	Notes
EDGARv 4.3.2 [4]	Waste	16	16	17	17	17	17		Includes industrial waste and MSW. Incineration and open burning
CMIP6[5,6]	Waste	112	116	120	124	129	130		Includes industrial waste and MSW
Wiedinmyer et al., 2014 [7]	MSW	1413							Open burning of MSW
This study	MSW	133					150	242-308	Includes incineration and open burning of MSW

Table S4. Studies assessing global emissions from waste Tg/yr.

Study	Sector	year	PM2.5	BC	OC	CO	SO2	NOx	VOCs	Notes
EDGARv 4.3.2 [4]	Waste	2012	0.107	0.006	0.010	0.040	0.040	0.090	0.030	Emissions from solid waste disposal sites and waste incineration
CMIP6[5,6]	Waste	2015		0.7	4	40	0.5	6	7	Includes industrial waste and MSW
Eclipse_V5a[2]	MSW	2015	2.5	0.4	1	10	0.1	0.3	1.4	
Wiedinmyer et al., 2014	MSW	2010	6	0.6	5	37	0.5	4	7	Emissions from open burning of MSW. VOC identified.
This study	MSW	2015	2.3	0.17	1.4	11	0.08	0.4	2.2	Includes incineration and open burning of MSW
Eclipse_V5a [2]	Total	2010	110	10	33	511	85	89	104	Estimates for PM2.5, BC, OC represent global total, whereof about 52% anthropogenic. Other pollutants refer to anthropogenic
CMIP6[5,6]	Total	2015		10	35	934	94	156	227	Total global emissions

S4. Description of the SSPS.

The Shared Socioeconomic Pathways (SSPs) provide five plausible pathways about probable world's socioeconomic development. Each SSP is accompanied by a narrative and a quantification of development [9]. A short description of the narratives in terms of economic development and demographics for each SSPs is presented below (see ref[10]):

- SSP1 'Sustainability': Economic growth is moderately high in developing countries leading to a reduction of inequality within and between countries. Low material growth and resource use. Current high fertility countries move towards low population while in low fertility countries there is an increase of fertility rates. Urbanization is still high in developing countries, the negative effects associated with it are limited.
- SSP2 'Middle of the road': Moderate economic growth. Income distribution shows an improvement but still inequalities are observed. Population growth is moderate, and urbanization is consistent with the historical trend.
- SSP3 'Regional Rivalry': Little economic growth due to lack of investment in education and technology. High inequalities within and between countries. Population growth is high, and urbanization slow.
- SSP4 'Inequality': Medium economic growth in high- and middle-income countries while low-income countries are far left behind. Thus, reflected in the high and low consumption patterns of the respective economies. Industrialized countries depict low fertility rates and population growth. In low-income countries urbanization is high forming urban-slums.
- SSP5 'Fossil Fueled Development': Income inequality decreases within regions and per capita income increases at a global level. Global population declines. Regions reach high level of urbanization.

Table S5. Description of the scenarios

Scenario	Socio-economic aspects	Environmental policy	Circular MSW management systems (MFR)
SSP1	Low population growth<br>Declining inequality<br>Global cooperation	Strong environmental policy	Food and plastic waste reduction of 50% by the year 2030<br>Global implementation of circular MSW management systems<br>Air pollution and GHGs emissions caused by inappropriate waste management are avoided.
SSP2	Moderate economic growth<br>Moderate population growth	Slow progress in achieving environmental targets and SDGs.	High income regions implement circular MSW successfully. Middle income regions implement the systems at a slow pace. All other countries improve their MSW management systems but are still left behind in terms of implementation.
SSP3	High population growth<br>Slow economic growth in developing countries	Environmental concerns not a priority	High income regions implement circular MSW successfully. Middle income regions implement the systems at a slow pace. All other countries are left far behind. Disparities between waste management in urban and rural areas are notorious.
SSP4	Medium economic growth in high- and middle-income countries.<br>Low economic growth in low-income countries<br>Social cohesion degrades	Environmental priority for the few affluent	High income regions implement circular MSW successfully. Middle income regions implement the systems at a slow pace. All other countries struggle to cope with the large quantities of waste generated.
SSP5	Rapid economic growth<br>Global population declines	Low regard for environment and SDGs	Global implementation of circular MSW management systems<br>No measures to reduce MSW generation<br>High air pollution controls
ECLIPSE_V6b	The rate of growth in population slows over time<br>Global economic activity continues to strengthen	Some progress in achieving environmental targets and SDGs.	Global implementation of circular MSW management systems<br>No measures to reduce MSW generation<br>High air pollution controls

S5. Abbreviations

Table S6. Abbreviations

BC	Black Carbon
CE	Circular Economy
CH_4	Methane
CLE	Current Legislation
CO	Carbon Oxide
CO_2	Carbon Dioxide
EF	Emission Factor
EU	European Union
GAINS	Greenhouse Gas- Air pollution Interaction and Synergies model
GDP	Gross Domestic Product
GHG	Greenhouse Gases
IAMs	Integrated Assessment Models
IEA	International Energy Agency
IPCC	Intergovernmental Panel on Climate Change
MFR	Maximum Technically Reduction
MSW	Municipal Solid Waste
NO_x	Nitrogen Oxide
OC	Organic Carbon
PM2.5	Particulate Matter <= 2.5 µg
SDGs	Sustainable Development Goals
SO_2	Sulfur Dioxide
SSPs	Shared Socioeconomic Pathways
UN	United Nations
VOCs	Volatile organic compounds
WEO	World Energy Outlook

S6. GDP per capita and share of urban population.

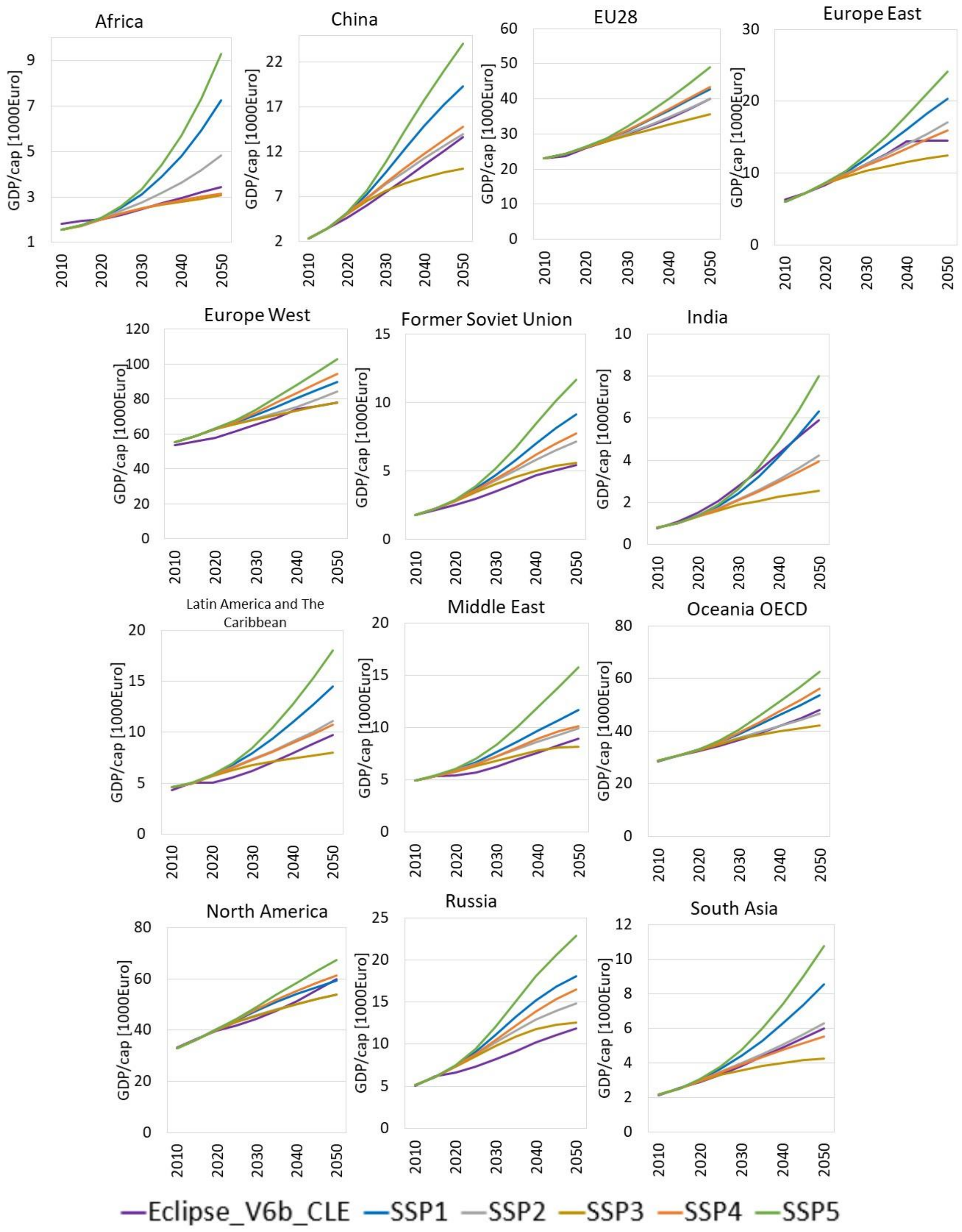

Fig. S2 . GDP per capita by region

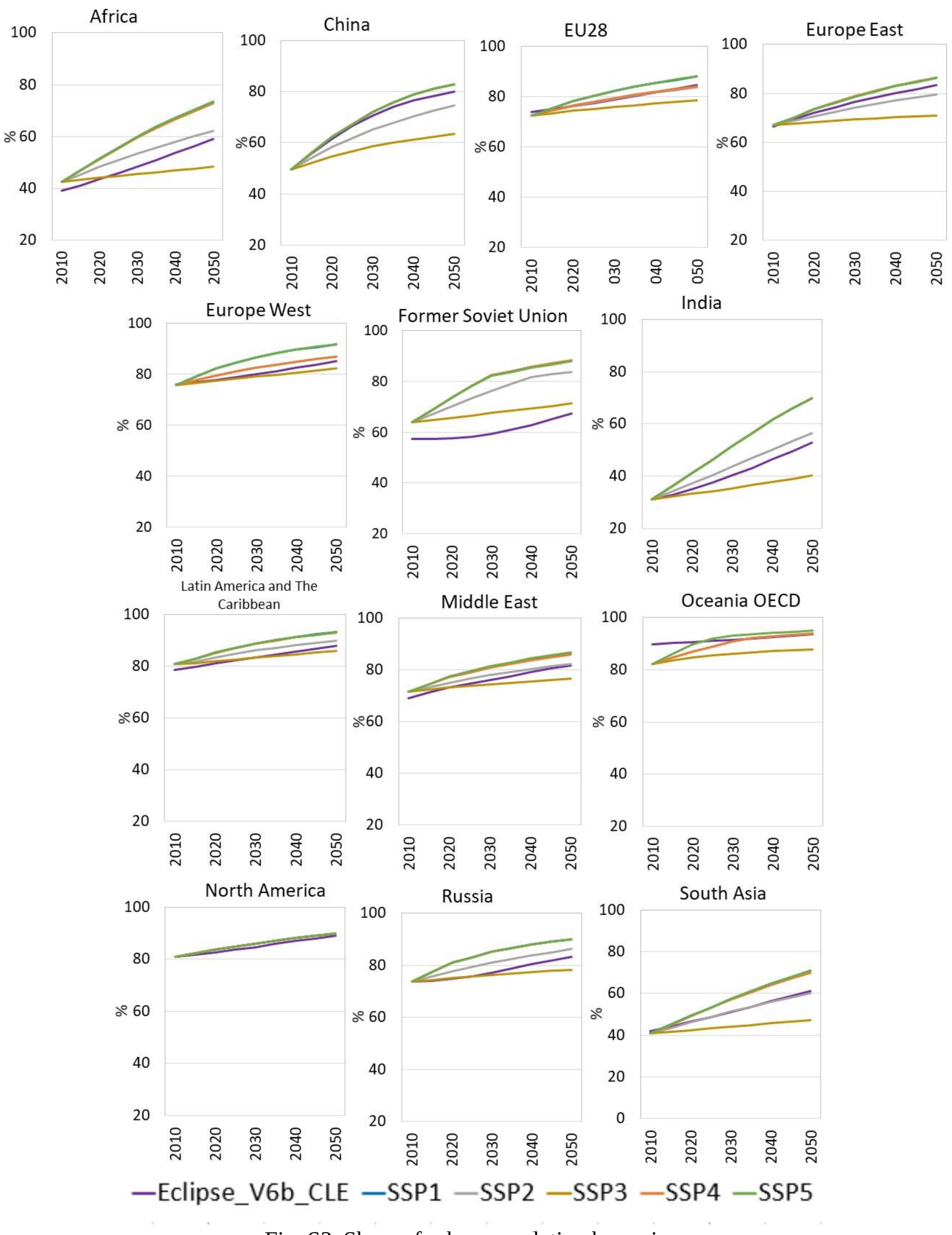

Fig. S3. Share of urban population by region.

S7. Description of the methodology to project municipal solid waste generation and composition.

A new methodology to project municipal solid waste generation and waste composition by income group was developed based on the assumption that average national waste generation rate and composition vary depending on the average national income level ref[11–13] [12]. Numerous studies [12,14–16] indicate that composition of municipal solid waste depends on socio-economic characteristics, geographical location and environmental features. Paper and plastic wastes are the main fractions of MSW in high-income countries, while food waste dominates in low income countries [12]. A panel data analysis is performed to determine the elasticity of the different variables on the generation of municipal solid waste per capita. The driver to project future municipal solid waste generation is GDP per capita (urbanization rate was also included as an explanatory variable, however, it has shown to be insignificant in all cases). Furthermore, since waste composition influences the carbon content and hence the material and energy recovery potential, projections of waste composition are needed. For future years, the composition of waste is recalculated based on an estimated elasticity of per capita food waste to GDP per capita. After projecting the future generation of food waste per capita, other types of waste are projected to make up the rest of total per capita MSW generated with the relative contribution of non-food waste in 2015 kept constant in future years.

Description of the variables and data to estimate MSW generation elasticities: Three different variables are used to run the panel analysis, namely, historical municipal solid waste generation per capita, gross domestic product per capita and urbanization rates. All variables are specified in logarithmic form in order to provide parameter estimates that can be directly interpreted as elasticity values. In total, the unbalanced panel data set comprises 1006 observations. In order to control for the influence of population growth, waste generation per capita is chosen instead of total waste generation as dependent variable in elasticity estimations [17]. Data on historical municipal solid waste generation in kilogram per capita are obtained from different sources (Table 1). The dataset for EU28 countries covers from 1995 to 2017, for some OECD countries the data covers between 5 and 31 years (e.g., Japan and South Korea).

Table S7. Urban-rural MSW generation per capita ratio

Country	Years	Waste generation data - Source
EU 28 countries	1995-2017	Eurostat (retrieved 2020) Table [env_wasmun]. Ireland: Environmental protection Ireland https://www.epa.ie/nationalwastestatistics/irelandswastestory/ Finland: Statistics Finland/Waste statistics, Finnish Environment Institute (SYKE)
Norway	1995-2017	Waste statistics from statistics Norway https://www.ssb.no/en/avfkomm
Switzerland	1995-2013	Eurostat (retrieved 2020) Table [env_wasmun]
Australia	2006-2015	OECD (retrieved 2020) Table [Municipal waste] and Australian Bureau of Statistics -waste accounts https://www.abs.gov.au/statistics/environment/environmental-management/waste-account-australia-experimental-estimates/2018-19
Japan	1985-2016	OECD (retrieved 2020) Table [Municipal waste]
South Korea	1985-2016	OECD (retrieved 2020) Table [Municipal waste]
Mexico	1991-2012	OECD (retrieved 2020) Table [Municipal waste]
New Zealand	2002-2017	OECD (retrieved 2020) Table [Municipal waste]
United States of America	1990- 2015	Advancing Sustainable Materials Management: 2018 Tables and Figures Assessing Trends in Materials Generation and Management in the United States November 2020. https://www.epa.gov/facts-and-figures-about-materials-waste-and-recycling/advancing-sustainable-materials-management
Brazil	2000-2012	OECD (retrieved 2020) Table [Municipal waste]
Chile	2000-2016	OECD (retrieved 2020) Table [Municipal waste]
Israel	2000-2017	OECD (retrieved 2020) Table [Municipal waste]
Colombia	2003-2011	SSPD 2011, OECD (retrieved 2020) Table [Municipal waste]
Russia	1999-2011	OECD (retrieved 2020) Table [Municipal waste]
Turkey	1995-2017	Eurostat (retrieved 2020) Table [env_wasmun]
Serbia	2006-2017	Eurostat (retrieved 2020) Table [env_wasmun]
Macedonia	2008-2017	Eurostat (retrieved 2020) Table [env_wasmun]
Malaysia	1996-2000	Department of statistics Malaysia (accessed 2016)
Kenya	1998-2009	
Montenegro	2008-2017	Eurostat (retrieved 2020) Table [env_wasmun]
Bosnia and Herzegovina	2008-2017	Eurostat (retrieved 2020) Table [env_wasmun]
Kosovo	2015-2017	Eurostat (retrieved 2020) Table [env_wasmun]
Peru	2012-2015	Municipalidad Metropolitana de Lima (MML) 2015

*Eurostat database[18] OECD database[19]

Data on Gross Domestic Product per capita in constant 2010 US dollars was obtained from the World development Indicators (World Bank, retrieved 2020). Urban population information was obtained

from EUROSTAT Table [EU-SILC survey [ilc_lvho01]] for EU28 and from the World Development Indicators (retrieved 2020) for other countries. To get an agreement between the dataset and guarantee consistency some adjustments on the information were needed due to the different definition of urbanization.

Elasticity estimation models: Historical data on municipal solid waste generation per capita (dependent variable) are plotted against GDP per capita (independent variable) in order to visualize the relationship between the two variables and to identify possible clusters of municipal waste generation (Fig. S4).

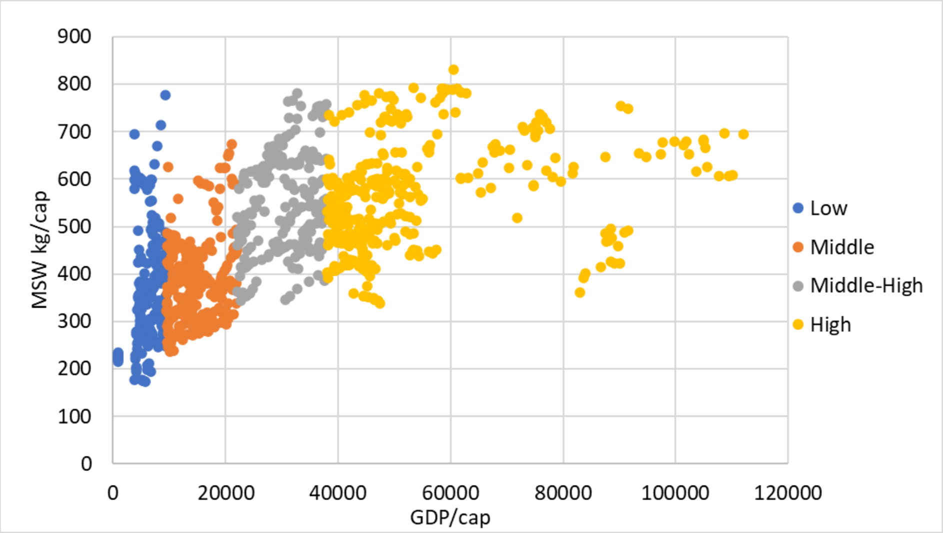

Fig. S4. Municipal solid waste vs GDP per capita.

The definition of the different income groups was carried out based on the distribution of the scatterplot. Note that in the subsequent projections, countries may over time move out of their initial income group into a higher income group following an increase in the GDP per capita. Hence, the group distribution of the municipal solid waste generation is dynamic over time. It is important to notice that this income group definition is independent than that of the World Bank or any other income classification. The income group definition here is specifically related to MSW generation.

The different income groups are classified as follows (GDP in constant 2010 US$): Low-income group is formed by countries/regions with GDP per capita lower than 9500 US$/year, middle-income group represents countries/regions with GDP per capita higher-equal than 9500 US$/year and lower than 22000 US$/year; middle-high income group represents countries/regions with GDP per capita higher-equal than 22000 US$/year and lower than 38000 US$/year and high-income group is formed by countries/regions with GDP per capita higher equal than 38000 US$/year. The latter group was then carefully revised as evidence has shown that some countries have already implemented some waste prevention programs. Fact that could interfere in the relationship between the variables. Therefore, a subgroup of countries with GDP per capita higher-equal than 38000 US$/year and years before

implementation of any waste prevention program was selected. At EU level, the Waste Framework Directive[1] requires Members States to adopt waste prevention programmes by December 2013. Therefore, the selection of the observations was done after reviewing the reported information in terms on MSW generation to EUROSTAT but also official national sources, together with a careful revision of the annual review progress in the completion and implementation of the programmes carried out by the European Environment Agency (EEA) in 2019[2]. For countries outside the EU (i.e., Japan), a similar process was carried out in which reported values were contrasted to the implementation of strategies and regulations to reduce MSW generation.

Furthermore, since waste composition influences on the one hand emissions of air pollutants and greenhouse gases and on the other hand, the circularity of resources, projections of waste composition are relevant. In particular, low-income countries tend to have a considerably higher fraction of food waste in the total municipal waste generated than high income countries. Therefore, changes in the future composition of waste are projected by income group based on an estimated elasticity of food waste generation to GDP per capita. Historical data on food waste generation is taken from ref[20]. The dataset comprises 882 observations in total. The elasticity is estimated for the same income groups as MSW in unbalanced panels. The panel data analysis is performed to determine the elasticity of the different variables on the generation of municipal solid waste per capita. Pooled OLS, fixed effects and random effects estimator models are run to test the effects of the explanatory variables on municipal waste generation per capita. In the pooled models a single slope is calculated for all countries and the between (cross-sectional) and within (time) variances are bluntly added up. When the cross-sectional variance is eliminated and the slopes are based on time variance only, the model is denoted a within estimator whereas in between models the time variance is eliminated and only cross-sectional variance is considered in the elasticity parameter. In fixed effect models, the within estimator is describing the slope while the country-specific effects are captured as country-specific constants. Finally, random effect model treats the individual effects as random variables and the variance is a weighted average of within and between variance [21]. Three different tests are applied to select the appropriate model. A Lagrange Multiplier (LM) test is applied to test for the cross-sectional dependence in heterogeneous panels (test random effects vs pooling). An F test is used to test for individual effects based on the comparison between the within and the pooling model and a Hausman test is used to evaluate the difference in vector coefficients between the fixed and random effects models. The results of the elasticity estimations of municipal solid waste generation to GDP per capita and urbanization rate and elasticity estimations of food waste generation (fraction in MSW) to GDP per capita are presented in Table S

[1] Directive 2008/98/EC, Article 29.
[2] https://www.eea.europa.eu/themes/waste/waste-prevention/countries/folder_contents?pagenumber=2&pagesize=20

Table S8. MSW generation elasticities to GDP per capita and urbanization rate

Variable kg/cap	Income group USD2010/cap	n	Number of observations	Explanatory variable	OLS	Fixed Effect	Random Effect	LM - test	Hausman - test
MSW	<9500	23	166	Constant	0.001		0.015	8.835	23.012
				GDP per capita	0.000	0.375***	0.008		
				Urbanization rate	0.013	0.003	0.011		
				R-square	-0.010	-0.001	-0.008		
	<9500	23	166	Constant	-0.001		0.013	8.923	23.643
				GDP per capita	0.000	**0.375*** **	0.007		
				Urbanization rate					
				R-square	-0.006	**0.006**	0.446		
	<9500	23	166	Constant	0.001		0.010	6.692	0.063
				GDP per capita					
				Urbanization rate	0.013	0.009	0.011		
				R-square	-0.004	-0.161	-0.004		
FW	<9500	23	166	Constant	-0.023***		5.033***	25.425	55.065
				GDP per capita	-0.002	**0.176*** **	0.161***		
				Urbanization rate					
				R-square	0.006	**0.473**	0.681		
MSW	>=9500 - <22000	18	253	Constant	-0.024**		-0.023~	2.351	2.438
				GDP per capita	0.250***	0.175**	0.224***		
				Urbanization rate	-0.504~	-0.127	-0.357		
				R-square	0.080	-0.032	0.060		
	>=9500 - <22000	18	253	Constant	-0.046***		**-0.022**	34.273	1.818
				GDP per capita	0.364***	0.160**	**0.183*** **		
				Urbanization rate					
				R-square	0.085	-0.028	**0.058**		
	>=9500 - <22000	18	253	Constant	-0.016*		-0.015	3.286	1.054
				GDP per capita					
				Urbanization rate	0.291	0.551*	0.437~		
				R-square	0.002	-0.059	0.008		
FW	>=9500 - <22000	18	253	Constant	**0.004*** **		5.052***	1.002	0.001
				GDP per capita	**0.133*** **	0.130***	0.130***		
				Urbanization rate					
				R-square	**0.628**	0.521			
MSW	>=22000 - <38000	22	201	Constant	-0.011		-0.021	8.378	0.368
				GDP per capita	0.204***	0.258**	0.241***		
				Urbanization rate	-0.263	-0.042	-0.104		
				R-square	0.096	-0.061	0.078		
	>=22000 - <38000	22	201	Constant	-0.011		**-0.021**	8.616	0.174
				GDP per capita	0.186***	0.254***	**0.233*** **		
				Urbanization rate					
				R-square	0.090	-0.055	**0.081**		
	>=22000 - <38000	22	201	Constant	-0.007		-0.022	7.436	9.389
				GDP per capita					
				Urbanization rate	-0.027	0.312	0.088		
				R-square	-0.005	-0.115	0.006		
FW	>=22000 - <38000	22	201	Constant	-0.023*		5.164***	4.513	0.108
				GDP per capita	-0.232***	-0.023	-0.036		
				Urbanization rate					
				R-square	0.083	-0.123	0.826		

Variable kg/cap	Income group USD2010/cap	n	Number of observations	Explanatory variable	OLS	Fixed Effect	Random Effect	LM - test	Hausman - test
MSW	>=38000*	16	230	Constant	-0.001		-0.001	0.051	0.024
				GDP per capita	0.536***	0.537 ***	0.536***		
				Urbanization rate	0.027	0.019	0.027		
				R-square	0.8971	0.256	0.303		
	>=38000*	16	230	Constant	-0.001		-0.001	0.051	0.002
				GDP per capita	0.539***	0.539***	**0.539*** **		
				Urbanization rate					
				R-square	0.307	0.259	**0.307**		
	>=38000*	16	230	Constant	-0.003		-0.003	0.043	0.068
				GDP per capita					
				Urbanization rate	0.473**	0.488**	0.473**		
				R-square	0.027	-0.041	0.027		
FW	>=38000	16	230	Constant	0.000		5.120***	6.681	3.611
				GDP per capita	-0.365***	0.056	**0.051**		
				Urbanization rate					
				R-square	0.182	-0.054	0.298		
MSW	All income groups	50	892	Constant	-0.004		-0.001	0.046	0.051
				GDP per capita	0.100***	0.099***	0.100***		
				Urbanization rate	0.016	0.016	0.016		
				R-square	0.025	-0.033	0.025		
	All income groups	50	892	Constant	-0.004		**-0.004**	0.046	0.048
				GDP per capita	0.103***	0.101***	**0.103*** **		
				Urbanization rate					
				R-square	0.001	-0.032	**0.025**		
	All income groups	50	892	Constant	-0.006		-0.006	0.061	0.010
				GDP per capita					
				Urbanization rate	0.042	0.002	0.042		
				R-square	0.001	-0.057	0.010		
FW	All income groups	46	927	Constant	-0.002		5.102***	13.507	5.999
				GDP per capita	0.099***	**0.101*** **	0.085***		
				Urbanization rate					
				R-square	0.033	**-0.013**	0.249		

Where:, $\varepsilon_{it}=u_i+v_{it}$ is an error term which is separated into an individual effects term and a residual omitted variables term, and ε_{it}~IID $(0,\sigma_\varepsilon^2)$ is an error term which are assumed to be normally distributed with mean zero and constant variance. * before implementation of waste prevention programmes or policies to reduce MSW generation.

S8. Rural-urban waste generation ratio

Table S9. Urban-rural MSW generation per capita ratio

Region	Rural Urban ratio	Comments	Source
Africa	0.53	Ratio between average kg/cap/year between North Africa (442) and Sub-Saharan Africa (237)	[22]
China	0.8/0.55	Based on average rural waste generation rate of 0.95 kg/cap/day for the year 2010 for provinces with high urban areas and 0.55 for provinces with lower urban areas.	[23]
Latin America and the Caribbean	0.56	Urban solid waste generation in LAC reaches between 0.6 to 0.93 kg/cap/day. Ratio between countries highly urbanized and less urbanized in Latin America.	[24]
EU15	0.8		
EU13	0.6	Based on a study carried out in Romania in which average waste generation rate of 0.4 kg/cap/day is stipulated. However, in peri urban areas waste generation rates are close to the ones in urban areas.	[25,26]
North America	0.8	Based on economic differences between urban and rural areas in the US.	[27]
Europe West	0.8	Assumed to similar to EU15	
Russia	0.6	Assumed to be similar to EU13	
Former Soviet Union	0.6	Assumed to be similar to EU13	
Middle East	0.6	Based on the reported MSW generation rates per capita for countries such as Mauritania, Morocco, Algeria, Tunisia, Egypt, Lebanon, Syria, Jordan, and Yemen.	[28]
Oceania	0.8		
India	0.55	Value based on MSW generation rates for different income levels, and specific reported data on urban/rural generation in Andhara Pradesh, Chandigarh, Kerala and Tamil Nadu.	[29–32]
South Asia	0.5	Value based on a study carried out in Thailand on the different MSW generation rates in different household types.	[33]

S9. Waste matrix in GAINS.

Table S10. Solid waste management technologies

Solid waste management technology	Municipal solid waste							
	Food	Glass	Metal	Other	Paper	Plastic	Textile	Wood
Open burned	X			X	X	X	X	X
Scattered and/or disposed to water-courses	X	X	X	X	X	X	X	X
Unmanaged solid waste disposal site - low humidity - < 5m deep	X			X	X		X	X
Unmanaged solid waste disposal site - high humidity - > 5m deep	X			X	X		X	X
Compacted landfill	X	X	X	X	X	X	X	X
Covered landfill	X			X	X		X	X
Landfill gas recovery and flaring	X			X	X		X	X
Landfill gas recovery and used	X			X	X		X	X
Low quality burning of waste	X			X	X	X	X	X
Incineration (poor air quality controls)	X			X	X	X	X	X
Incineration (high quality air pollution controls - energy recovery)	X			X	X	X	X	X
Anaerobic digestion	X							
Composting	X							
Recycling		X	X		X	X	X	X

S10. MSW management narratives and regional aggregation.

Table S11. MSW management narratives

Scenario	Description
SSP1_MFR	Maximum municipal food waste reduction of 50% by the year 2030 based on Lipinski et al., 2013[34] and based on the target adopted by the United Nations Assembly in 2015 of halving per capita food waste at the retail and consumer level as a part of the 2030 Sustainable Development Goals.<br>A maximum municipal plastic waste rate reduction of 50% by the year 2030 as a part of the 2030 Sustainable Development Goals.<br>Waste management policies are implemented at a global level resulting in an improvement of waste management systems.<br>Waste technology transfer and capacity building is facilitated allowing the less favored countries to improve and develop appropriate waste management systems in both urban and rural areas. Hence, environmental impacts such as air pollution and GHGs emissions caused by inappropriate waste management are avoided.
SSP2_MFR	EU28, EU West, EU East, Oceania and North America regions continue developing and implementing policies to meet the proposed environmental targets related to waste.<br>Russia and the Former Soviet Union countries also implement similar policies but a slower pace.<br>All other countries either continue or start developing strategies to improve their waste management systems but are still left behind in terms of implementation.
SSP3_MFR	EU28, EU West, EU East, Oceania, and North America countries continue developing and implementing policies to meet the proposed environmental targets related to waste.<br>Russia and The Former Soviet Union countries also implement similar policies but a slower pace.<br>All other countries are left far behind due to a lack of international support in terms of technology transfer and capacity building. Environmental concerns related to waste are not a priority in these countries. Disparities between waste management in urban and rural areas are notorious.
SSP4_MFR	EU28, EU West, EU East, Oceania, and North America and Russia and The Former Soviet Union countries continue developing and implementing policies to meet the proposed environmental targets related to waste.<br>Russia and The Former Soviet Union countries catch up with European countries in terms of waste management.<br>All other countries continue struggling to cope with the large quantities of waste generated.

SSP5_MFR	Waste technology transfer and capacity building is facilitated allowing the less favored countries to improve and develop appropriate waste management systems in both urban and rural areas. However, policies targeted to waste reduction are still missing.
Eclipse_V6b_MFR	Waste management systems are improved at a global level. There is collaboration between and within nations. Reduction of environmental impacts caused by waste management are successfully implemented.

Table S5. Regional Aggregation

Income group	Country/region
Africa	South Africa, Tanzania, Egypt, Kenya, Nigeria, North Africa (includes Algeria, Morocco, Libya, Tunisia, Sudan), East Africa, Western Africa, Rest Africa
China	Anhui, Beijing, Chongqing, Fujian, Gansu, Guangdong, Guangxi, Guizhou, Hainan, Hebei, Heilongjiang, Henan, Hong Kong and Macau, Hubei, Hunan, Jilin, Jiangsu, Jiangxi, Liaoning, Inner Mongolia, Ningxia, Qinghai, Shaanxi, Shanghai, Sichuan, Tianjin, Tibet, Xinjiang, Yunnan and Zhejiang
EU28	Austria, Belgium, Bulgaria, Cyprus, Croatia, Czech Republic, Denmark, Finland, France, Germany, Ireland, Luxembourg, Netherlands, Sweden, Greece, Malta, Portugal, Slovenia, Estonia, Hungary, Latvia, Lithuania, Poland, Romania, Slovak Republic, Italy, Spain, United Kingdom.
EU-East	Albania, Bosnia and Herzegovina, Kosovo, Macedonia, Montenegro, Serbia, Turkey.
EU-West	Norway, Iceland, Switzerland
Former Soviet Union (FSOV)	Armenia, Former Soviet Union States (includes Tajikistan, Turkmenistan, and Uzbekistan), Georgia, Azerbaijan, Kazakhstan, Belarus, Moldova, Kyrgyzstan
India	Andhra Pradesh, Assam, West Bengal, Bihar, Chhattisgarh, Delhi, North East (excl Assam), Goa, Gujarat, Haryana, Himachal Pradesh, Jharkhand, Karnataka, Kerala, Maharashtra, Manipur, Orissa, Punjab, Rajasthan, Tamil Nadu, Uttarakhand, Uttar Pradesh, Jammu Kashmir
Latin America and The Caribbean	Argentina, Caribbean (includes countries in the Caribbean region), Chile, Brazil, Mexico, Central America, Colombia, Ecuador, Bolivia, Paraguay, Perú, Uruguay, Venezuela, and Other Latin America.
Middle East	Middle East, Iran, Israel, Saudi Arabia
North America	United States and Canada.
Oceania	Australia, New Zealand, Japan
Russia	Russia (Europe – Asia)
South Asia	Afghanistan, Bangladesh (Dhaka and rest of Bangladesh), Cambodia, North Korea, South Korea, Myanmar, Taiwan, Nepal, Pakistan (Karachi, NW frontier provinces Baluchistan, Punjab and Sindh), Philippines (Bicol, Luzol and Manila), Sri Lanka, Thailand (Bangkok, Central Valley, North Eastern Plateau, Northern Highlands and Southern Peninsula), Vietnam (North and South).

References

1. Anenberg, S. *et al.* Global air quality and health benefits of mitigating short-lived climate forcers. (2011).
2. Klimont, Z. *et al.* Global anthropogenic emissions of particulate matter including black carbon. *Atmospheric Chemistry and Physics* **17**, 8681–8723 (2017).
3. Reyna-Bensusan, N. *et al.* Experimental measurements of black carbon emission factors to estimate the global impact of uncontrolled burning of waste. *Atmospheric Environment* **213**, 629–639 (2019).
4. Crippa, M. *et al.* Gridded emissions of air pollutants for the period 1970–2012 within EDGAR v4.3.2. *Earth System Science Data* **10**, 1987–2013 (2018).
5. Gidden, M. *et al.* Global emissions pathways under different socioeconomic scenarios for use in CMIP6: A dataset of harmonized emissions trajectories through the end of the century. *Geoscientific Model Development* **12**, 1443–1475 (2019).
6. Hoesly, R. M. *et al.* Historical (1750–2014) anthropogenic emissions of reactive gases and aerosols from the Community Emissions Data System (CEDS). *Geoscientific Model Development* **11**, 369–408 (2018).
7. Wiedinmyer, C., Yokelson, R. J. & Gullett, B. K. Global Emissions of Trace Gases, Particulate Matter, and Hazardous Air Pollutants from Open Burning of Domestic Waste. *Environ. Sci. Technol.* **48**, 9523–9530 (2014).
8. Höglund-Isaksson, L., Gómez-Sanabria, A., Klimont, Z., Rafaj, P. & Schöpp, W. Technical potentials and costs for reducing global anthropogenic methane emissions in the 2050 timeframe–results from the GAINS model. *Environmental Research Communications* **2**, 025004 (2020).
9. O'Neill, B. C. *et al.* A new scenario framework for climate change research: the concept of shared socioeconomic pathways. *Climatic Change* **122**, 387–400 (2014).
10. O'Neill, B. C. *et al.* The roads ahead: Narratives for shared socioeconomic pathways describing world futures in the 21st century. *Global Environmental Change* **42**, 169–180 (2017).

11. Höglund-Isaksson, L. *Global anthropogenic methane emissions 2005–2030: technical mitigation potentials and costs*. (2012) doi:10.5194/acpd-12-11275-2012.
12. Hoornweg, D. & Bhada-Tata, P. *What a waste. A global review of solid waste management*. (2012).
13. Kaza, S., Bhada-Tata, P. & Van Woerden, F. *What a waste 2.0. A global snapshot of solid waste management to 2050*. (2018).
14. UNEP & ISWA. *Global Waste Management Outlook*. (2015).
15. SWEEPNET. Regional profile on the solid waste management situation in Middle East and North Africa. (2012).
16. Wilson, D. C., Rodic, L., Scheinberg, A., Velis, C. A. & Alabaster, G. Comparative analysis of solid waste management in 20 cities. *Waste Manag Res* **30**, 237–254 (2012).
17. Lebersorger, S. & Beigl, P. Municipal solid waste generation in municipalities: Quantifying impacts of household structure, commercial waste and domestic fuel. *Waste Management* **31**, 1907–1915 (2011).
18. EUROSTAT database. Statistics | Eurostat (europa.eu)
19. OECD database. Waste - Municipal waste - OECD Data
20. Chen, D. M.-C., Bodirsky, B. L., Krueger, T., Mishra, A. & Popp, A. The world's growing municipal solid waste: Trends and impacts. *Environmental Research Letters* (2020).
21. Hsiao, C. *Analysis of Panel Data*. (Cambrige University Press., 1986).
22. Scarlat, N., Motola, V., Dallemand, J. F., Monforti-Ferrario, F. & Mofor, L. Evaluation of energy potential of Municipal Solid Waste from African urban areas. *Renewable and Sustainable Energy Reviews* **50**, 1269–1286 (2015).
23. Zeng, C., Niu, D. & Zhao, Y. A comprehensive overview of rural solid waste management in China. *Frontiers of Environmental Science & Engineering* **9**, 949–961 (2015).
24. Grau, J., Terraza, H., Rodríguez, V., Rihm, A. & Sturzenegger, G. Solid waste management in Latin America and the Caribbean. (2015).

25. Ciuta, S., Apostol, T. & Rusu, V. Urban and Rural MSW Stream Characterization for Separate Collection Improvement. *Sustainability* **7**, 916–931 (2015).

26. Mihai, F.-C. *Rural waste generation : a geographical survey at local scale*. (2016). doi:10.31235/osf.io/z7xpj.

27. EDR. Economic Differences: Urban and Rural Areas. (2017).

28. Ismail, A. Towards greeing the solid waste sector in the middle east and north africa region. (2012).

29. Feedback Infra Private Limited. *Detailed Project Report on Municipal Solid Waste Management for Visakhaptnam*. (2015).

30. Goverment of Tamil Nadu. *Data base on solid waste management in Tamil Nadu*. (2016).

31. Rajan, B., soorya, r, vincy, m & A.P., P. *Solid waste generation and associated problems of selected tourism destinations in Kerala - A comparative approach*. (2015).

32. Ravindra, K., Kaur, K. & Mor, S. System analysis of municipal solid waste management in Chandigarh and minimization practices for cleaner emissions. *Journal of Cleaner Production* **89**, 251–256 (2015).

33. Hiramatsu, A., Hara, Y., Sekiyama, M., Honda, R. & Chiemchaisri, C. Municipal solid waste flow and waste generation characteristics in an urban-rural fringe area in Thailand. *Waste management & research : the journal of the International Solid Wastes and Public Cleansing Association, ISWA* **27**, 951–60 (2009).

34. Lipinski, B. *et al.* Reducing food loss and waste. (2013).
